# Probing the interaction between NatA and the ribosome for co-translational protein acetylation

**DOI:** 10.1371/journal.pone.0186278

**Published:** 2017-10-10

**Authors:** Robert S. Magin, Sunbin Deng, Haibo Zhang, Barry Cooperman, Ronen Marmorstein

**Affiliations:** 1 Department of Biochemistry and Biophysics, Abramson Family Cancer Research Institute, Perelman School of Medicine, University of Pennsylvania, Philadelphia, Pennsylvania, United States of America; 2 Graduate Group in Biochemistry and Biophysics, Perelman School of Medicine, University of Pennsylvania, Philadelphia, Pennsylvania, United States of America; 3 Department of Chemistry, University of Pennsylvania, Philadelphia, Pennsylvania, United States of America; Saint Louis University School of Medicine, UNITED STATES

## Abstract

N-terminal acetylation is among the most abundant protein modifications in eukaryotic cells. Over the last decade, significant progress has been made in elucidating the function of N-terminal acetylation for a number of diverse systems, involved in a wide variety of biological processes. The enzymes responsible for the modification are the N-terminal acetyltransferases (NATs). The NATs are a highly conserved group of enzymes in eukaryotes, which are responsible for acetylating over 80% of the soluble proteome in human cells. Importantly, many of these NATs act co-translationally; they interact with the ribosome near the exit tunnel and acetylate the nascent protein chain as it is being translated. While the structures of many of the NATs have been determined, the molecular basis for the interaction with ribosome is not known. Here, using purified ribosomes and NatA, a very well-studied NAT, we show that NatA forms a stable complex with the ribosome in the absence of other stabilizing factors and through two conserved regions; primarily through an N-terminal domain and an internal basic helix. These regions may orient the active site of the NatA to face the peptide emerging from the exit tunnel. This work provides a framework for understanding how NatA and potentially other NATs interact with the ribosome for co-translational protein acetylation and sets the foundation for future studies to decouple N-terminal acetyltransferase activity from ribosome association.

## Introduction

N-terminal acetylation is one of the most abundant protein modifications. In eukaryotic cells, 50–90% of all soluble proteins are N-terminally acetylated [1]. The functional consequences of N-terminal acetylation are diverse at both the cellular and molecular level. N-terminal acetylation plays roles in apoptosis [2–4], gene regulation [5], protein localization [6–8], protein stability [9], and mediating protein-protein interactions [10–12]. The modification is necessary for development in a wide variety of organisms [13–16], and misregulation of N-terminal acetylation is implicated in numerous developmental disorders and cancers [17, 18], making N-terminal acetylation a potential therapeutic target.

N-terminal acetylation is carried out by a family of N-terminal acetyltransferases (NATs), which differ in their substrate specificity [1]. NatA, B, and C are responsible for acetylating the vast majority of N-termini in the cell. Each of these NATs has hundreds of substrates and recognizes N-termini based primarily on the first two residues [19]. NatA recognizes N-termini with small residues (Ala, Cys, Gly, Ser, Thr, Val) at their N-terminus after the initial methionine (iMet) has been cleaved by methionine aminopeptidase [20]. NatB acetylates substrates with an iMet followed by an acidic residue or their corresponding amides, and NatC acetylates N-termini with an iMet followed by large hydrophobic residues [21]. NATs A, B and C are comprised of a catalytic subunit (Naa10, 20, or 30 for NatA, B, and C, respectively) and auxiliary subunit (Naa15, 25 or 35), and require at least both of these subunits for catalytic activity [1]. Some NATs, like NatA, have additional binding partners. These can be other NATs, like Naa50 [22], or regulatory proteins like HYPK [23]. The other NATs are more specialized in their substrates and localization. Naa40 is a highly specific NAT and only acetylates histones H2A and H4 [24, 25], and Naa60 is localized to the Golgi and primarily acetylates the N-termini of membrane proteins [26–28].

It has been long known that N-terminal acetylation is largely a co-translational process [22]. Experiments conducted over 30 years ago showed that nascent N-termini isolated from ribosomes were acetylated with as few as 25 residues translated [29–31]. More recently, NatA, B and C have all been shown to interact with the ribosome through their auxiliary subunits in both yeast and human cells [32]. For NatA, this interaction is salt dependent, and a pull down from a yeast system indicated ribosomal proteins L23 (Rpl25p) and L29 (Rpl35p) in the NatA interaction [32]. Both of these proteins are found near the peptide exit tunnel in the 60S subunit of the ribosome. Moreover, N-terminal acetylation has also been shown to compete with signal recognition particle (SRP) targeting, further suggesting a dynamic interplay of various ribosomal binding proteins which are involved in N-terminal processing [33].

In addition to N-terminal acetylation, there are a number of other well-known co-translational processes and modifications [34]. These include translational stalling and localization to the endoplasmic reticulum by the SRP for transmembrane proteins, or those destined for the secretory pathway [35]. Other co-translational modifications include iMet removal by methionine aminopeptidases (MAP), methionine deformylation in bacteria by peptide deformylases (PDF), and N-terminal myristoylation by N-terminal myristoylases [34]. The two enzymatic processes that are most analogous to N-terminal acetylation are MAP and PDF activities. Both are very widespread, and occur on a high percentage of all proteins that are translated [36]. These two proteins both directly interact with the ribosome and are also both highly dynamic with a high on/off rate for association with the ribosome [37, 38].

In addition to these enzymatic events, there are a number of protein chaperones which bind to the emerging polypeptide at the ribosome exit tunnel to assist in co-translational folding [36]. These include trigger factor in bacteria [39, 40], NAC and the Ssb/Ssz/Zuotin triad in eukaryotes [41, 42]. Thus, the nascent N-terminus immediately encounters a number of factors involved in its processing as soon as it reaches the end of the exit tunnel. Importantly, all of these proteins are believed to directly interact with the ribosome. Although the detailed molecular basis for these interactions are not known for all of the factors, there do seem to be commonalities. The ribosomal protein L23 acts as an important docking site for SRP, trigger factor, NAC, and NatA, and many proteins use electrostatic interactions to interact with the ribosome, with both the rRNA and a conserved negatively charged patch found on a surface exposed region of L23 [39, 43–46].

Based on this previous research, we sought to probe the interaction between NatA and the ribosome, particularly in the structural determinants of NatA that mediate this interaction. We chose NatA, since it is the best characterized NAT both structurally and functionally [1, 23, 47]. By using the rationale that the NATs are likely to act similarly to MAP and PDF, we found that NatA also uses conserved, positively charged regions to interact directly with the ribosome in the absence of other stabilizing factors. These findings have implications for understanding how NatA and potentially other NATs interact with the ribosome for co-translational protein acetylation and will contribute to future studies to decouple NatA N-terminal acetyltransferase activity from ribosome association.

## Materials and methods

### *Schizosaccharomyces pombe* ribosome purification

Ribosomes were purified from *S*. *pombe* based on a modified preparation described for the *S*. *cerevisiae* ribosome [48]. *S*. *pombe* (strain 972 h-) was grown to an OD_600_ of 5 in YPD at 30°C. Cells were pelleted by centrifugation, re-suspended with YP (i.e. without glucose) and incubated in flasks with vigorous shaking (250 rpm) for 10 minutes. This glucose starvation step was done to ensure that the ribosomes were not translating protein, and would be in the apo form when purified. All further steps were performed at 4°C. Cells were pelleted by centrifuged and washed in buffer M (30 mM Hepes pH 7.5, 50 mM KCl, 10 mM MgCl_2_, 8.5% mannitol, 2 mM DTT, 0.5 mM EDTA). Cells were pelleted again, the supernatant was removed, and the cells were frozen in liquid nitrogen. The cells were lysed using a Mixer Mill MM 400 (Retsch) and the resulting cell powder was resuspended in buffer M and supplemented with one complete protease inhibitor tablet (without EDTA, Thermo), 100 μL RNasin, Pefablock (final concentration 2 mM) and Na-Heparin (final concentration 0.8 mg/ml) The resulting lysate was clarified by centrifugation (31,000g for 9 min). The supernatant was saved, and PEG 20,000 (Hampton Research) was added to a final concentration of 4.5% w/v and the solution was left to stand for 5 minutes on ice. The solution was clarified by centrifugation (20,000g for 5 min) and the supernatant was decanted to a new tube. Residual solution was “squeezed” out from the pellet by an additional short 1 min. centrifugation. The KCl concentration was then adjusted to 130 mM. After 5 min on ice, the PEG 20,000 concentrations were adjusted to 8.5% and the solution was left to stand for 10 min on ice. Ribosomes were precipitated (17,500g for10 min), the supernatant was discarded and residual solution was removed by a short spin of the pellet (14,500g for 1 min). The pellets were stored at -80°C until needed.

Ribosomes were suspended in buffer M2 (buffer M with KCl concentration adjusted to 150 mM and supplemented with protease inhibitors and heparin). Ribosomes were further purified by a 15–30% sucrose gradient in buffer A (20 mM Hepes-K pH 7.5, 120 mM KCl, 8.3 mM MgCl_2_, 2 mM DTT, 0.3 mM EDTA) using a VTi 50 rotor (Beckman) at 40,000 rpm for 1.5 h. After the appropriate fractions were collected based on A_260_ monitoring, KCl and MgCl_2_ concentrations were adjusted to 150 mM and 10 mM respectively, PEG 20% was then added to a final concentration of 7% w/v and the solution was left to stand 10 min. on ice. Ribosomes were precipitated (17,500g for 10 min), the supernatant was discarded, and residual solution was removed by a short spin of the pellet (14,500g for 1 min.). Ribosomes were suspended (20 mg/ml) in buffer G (10 mM Hepes pH 7.5, 50 mM KOAc, 10 mM NaCl, 2 mM DTT, 5 mM Mg(OAc)_2_). The concentrations of ribosomes were determined by the absorbance at 260 nm and using the extinction coefficient 5×10^7^ cm^-1^M^-1^ [49].

### Purification of NatA and enzyme assays

NatA was purified and assayed as described [47] with the following changes. After the protein was eluted from the nickel column, the His-tag was not cleaved off of the Naa15 subunit with TEV protease (Naa10 does not have a tag). After elution from the nickel column, the protein was directly dialyzed into ion exchange buffer containing 25 mM sodium citrate monobasic pH 5.5, 10 mM NaCl, and 1 mM βME. The subsequent ion exchange and gel filtration steps were performed as described [47].

Mutants of NatA were generated using standard site directed mutagenesis using the wild type NatA as a template. The purification of these mutants was identical to the wild type enzyme.

Acetylation assays were performed as described [47]. SASE refers to a NatA substrate, and MLGP refers to a NatE substrate. The first seven N-terminal residues of these peptides corresponds to protein substrates identified *in vivo* from proteomic studies [47], and the remaining residues contain a poly-Arginine track to ensure that the peptides adhere to the phosphocellulose used in the assays. The full peptides are: SASE- SASEAGVRWGRPVGRRRRP, and MLGP-MLGPEGGRWGRPVGRRRRP

### Sedimentation assays of NatA-ribosome complex

Sedimentation assays were carried out by combining ribosomes and NatA to final concentrations of 1 μM and 1.2 μM, respectively. 35 μl of this solution was added on top of 80 ul of Buffer G supplemented with 30% sucrose. Samples were centrifuged at 120,000 rpm in an S120-AT2 rotor (Thermo) in an ultracentrifuge. The supernatant was removed and ribosomal pellets were resuspended in 110 μl of Buffer G. Samples were run on SDS-PAGE to analyze the amount of NatA that co-sedimented with the ribosome. When necessary, a Western blot was performed using an anti-His antibody (GE Healthcare Life Sciences catalogue #27-4710-01, monoclonal from mouse, final dilution 1:2000) against the His-tag of the Naa15 subunit. For salt-sensitivity assays, the buffers were supplemented with the appropriate amount of KCl and performed as described above.

### K_d_ determination of NatA-ribosome association

Co-sedimentation assays were performed as above, with the following changes. A total of 120 μl of 30% sucrose was used, and 40 μl of sample was placed on top of that. A constant NatA concentration of 0.5 μM was used, and ribosome concentration varying from 0.1–11 μM was used. A western blot was performed, and the ratio of NatA in the pellet was quantified using ImageJ. The primary antibody dilution was 1:1000, and the secondary antibody dilution was 1:2500. The secondary antibody was Amersham ECL Mouse IgG, HRP-linked whole Ab from sheep (Product code NA931-ML). The resulting curve was fit using Prism, and the K_d_ was calculated using the equation Y = Kd+0.5+X-sqrt((Kd+0.5+X)^2-2X), where X is the concentration of ribosome in μM, and Y is the proportion of NatA in the pellet. This equation takes into account the non-negligible concentration of NatA and ribosome in the experimental set up. It also assumes a 1:1 stoichiometry of NatA:ribosome (see Results section for more discussion), and that all of the ribosomes in the sample can bind to NatA.

### Size exclusion chromatography assays

A total of 500 μl of NatA (6 μM) and ribosome (1.8 μM) was injected onto a superose 6 column and monitored with both 260 nm and 280 nm light. Appropriate fractions were run on SDS-PAGE and stained with colloidal coomassie blue for imaging.

## Results

### NatA mediates a salt-dependent interaction with the ribosome

**Fig 1:**
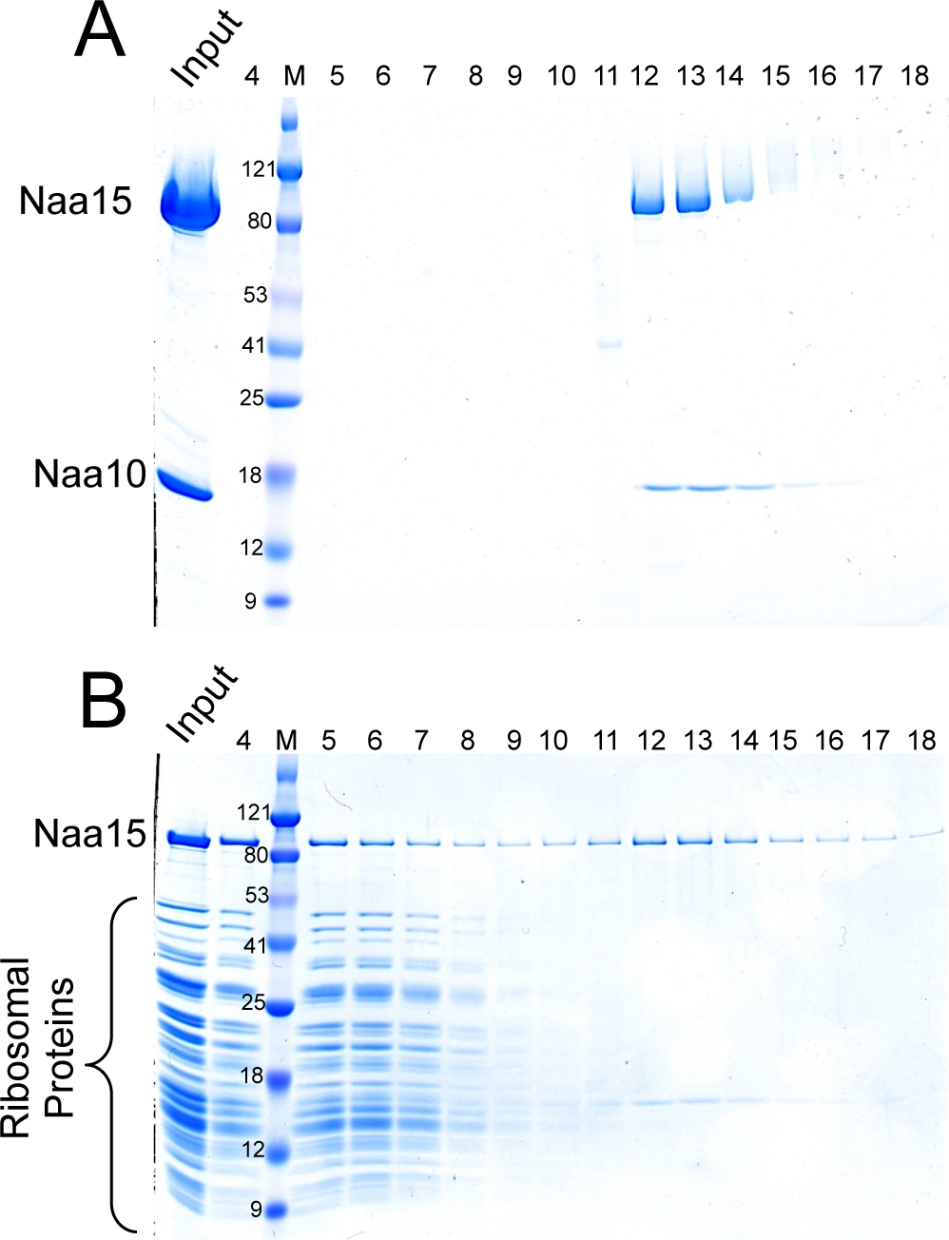
NatA binds to the ribosome in vitro. (A) Fractions of NatA eluting off of a Superose 6 column. The upper band is Naa15 and the lower band is Naa10. (B) Fractions of the ribosome and an excess of NatA eluting off of a Superose 6 column. Note the excess NatA eluting in fraction 12.

In order to study the NatA-ribosome interaction, we developed a purification for *Schizosaccharomyces pombe* ribosome, as the structure of *S*. *pombe* NatA is known and is readily recombinantly expressed in *E*. *coli* [47]. As mentioned, NatA consists of two subunits, Naa10, its catalytic subunit, and Naa15, its auxiliary subunit. We first tested whether NatA and the ribosome would co-elute from a Superose 6 size exclusion column as a stable complex. When run alone, NatA eluted from the column at fraction 12 (Fig 1A). However, when an excess of NatA is run with the ribosome, there is a clear shift in the position of the NatA, which coeluted with the ribosome earlier in the column. Importantly, the excess NatA began eluting around fraction 12 (Fig 1B), which is similar to the elution profile of NatA alone (Fig 1A). This suggests that the presence of NatA in the earlier fraction with the ribosome is not caused by ribosome-mediate aggregation of NatA. This result indicates that the purified NatA and ribosome can form a complex *in vitro* with no other mediating factors.

**Fig 2:**
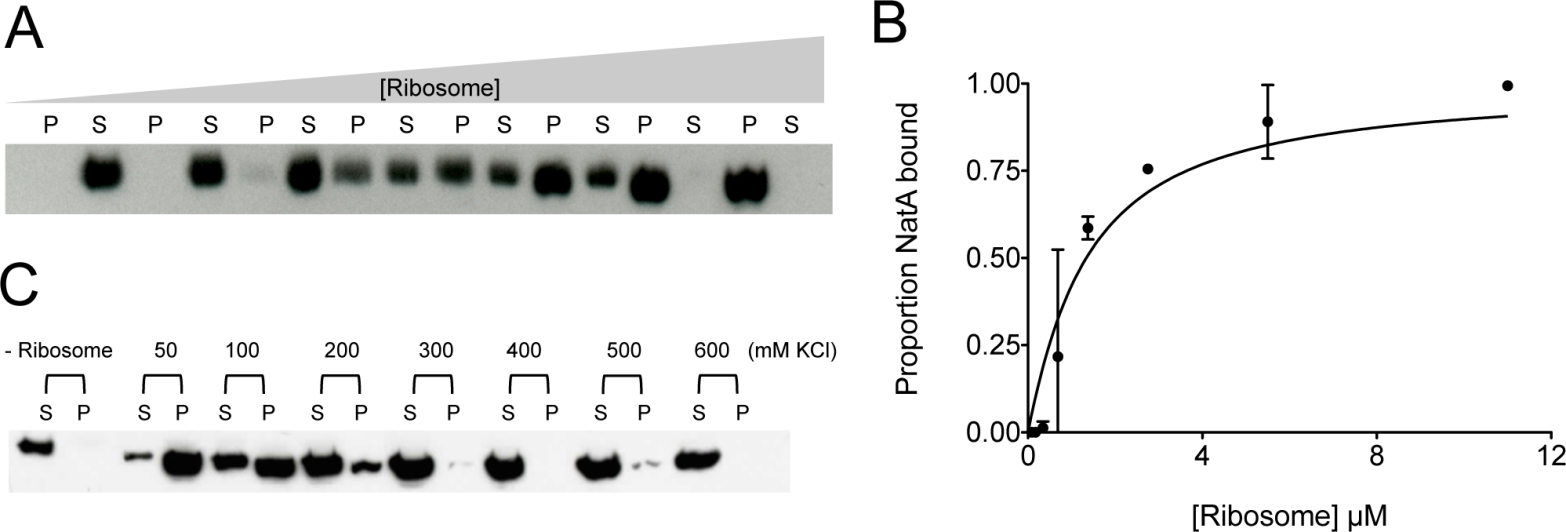
The NatA ribosome interaction has low μM affinity and is salt dependent. (A) Representative Western blot of NatA/ribosome co-sedimentation assay with increasing concentration of ribosome. P stands for pellet, and S stands for supernatant. The western blot targets the His tag on the Naa15 subunit (Naa10 is untagged) (B) An affinity curve of the ribosome NatA interaction quantified from the co-sedimentation assay. Assay was performed in duplicate (C) Co-sedimentation assay in increasing KCl concentrations. The first two lanes are NatA without ribosome present. S stands for supernatant and P stands for pellet. Uncropped gels are shown in S1 Fig.

We then performed co-sedimentation analysis of the NatA-ribosome complex. We first carried out a sedimentation assay with increasing concentration of ribosome to observe the binding profile of NatA and the ribosome (Fig 2A). We calculated the proportion of NatA in the pellet and supernatant fractions after spinning down in an ultracentrifuge. After fitting the curve, we found that the complex had a K_d_ of 1.1 ± 0.25 μM, which is similar to the calculated K_d_ of both methionine aminopeptidase [37], and the signal recognition particle [50], which are both in the low μM- mid nM range (Fig 2B).

Previous studies showed that the NatA-ribosome interaction is salt dependent *in vivo*. We performed the same analysis with purified NatA and ribosome. We found that the same held true in our experiment. As the salt concentration increased, the interaction weakened until NatA pull down by the ribosome was undetectable (Fig 2C).

### Two conserved electropositive regions in NatA are responsible for ribosome interaction

**Fig 3:**
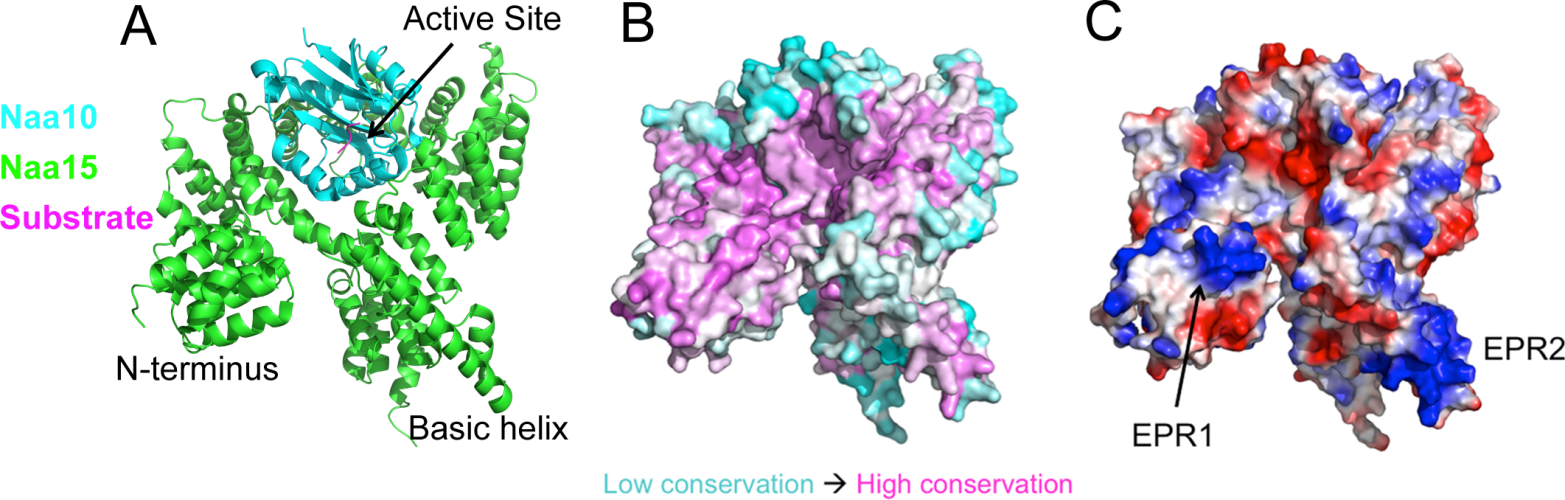
Conservation analysis and electrostatic surface of NatA show two regions important for ribosome binding. (A) A cartoon representation of the NatA complex. Naa10 is shown in cyan, Naa15 in green, and the peptide substrate in magenta. The N-terminus and internal basic helix are indicated, as is the active site where N-termini are acetylated. (B) Conservation map of the NatA complex. Magenta areas represent regions of high sequence conservation and cyan areas represent regions of low sequence conservation. (C) Electrostatic potential map of NatA. Blue areas represented regions which are electropositive, and red areas represent regions which are electronegative. Electropositive region 1 (EPR1), and electropositive region 2 (EPR2) are indicated.

**Fig 4:**
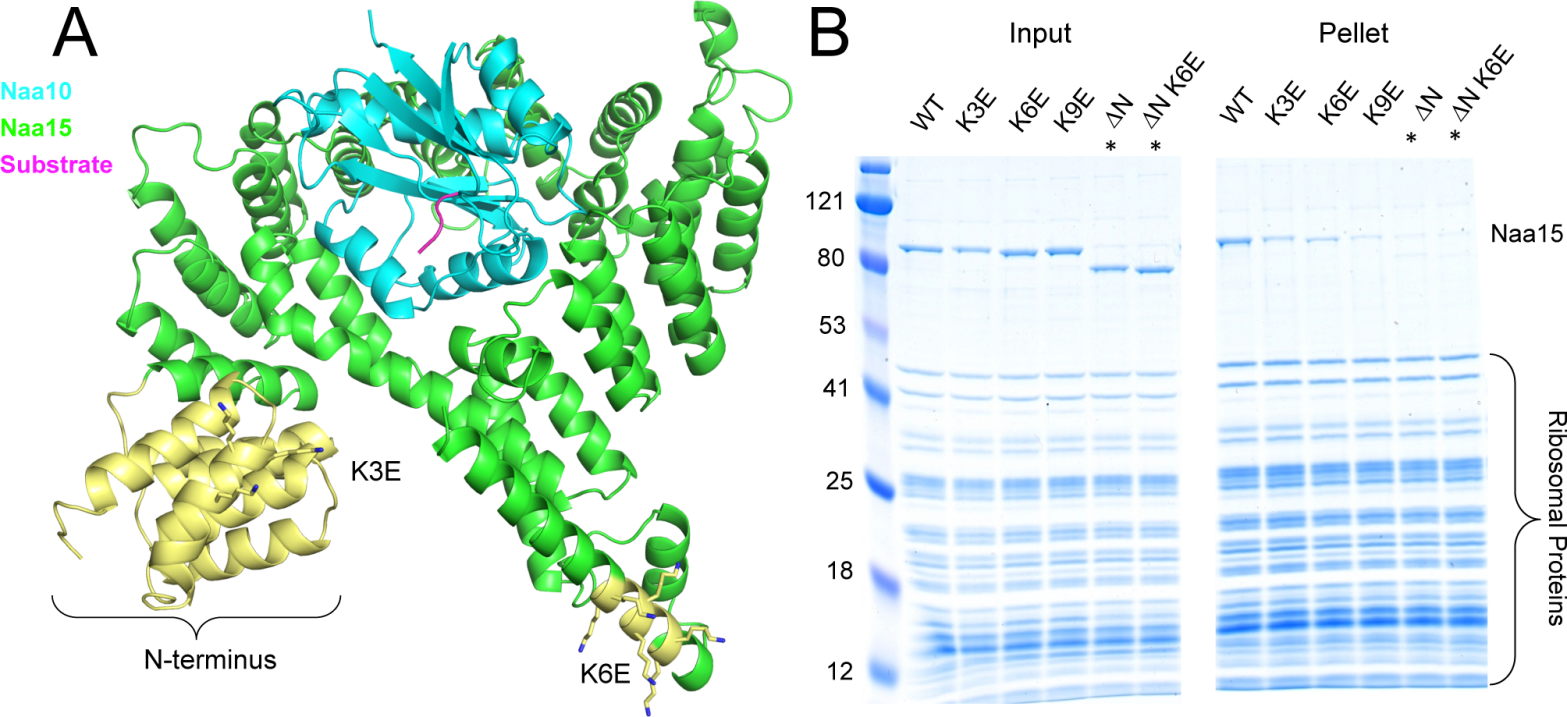
Pull down analysis of NatA mutants. (A) Location of NatA mutants used in this study. Mutated regions are shown in yellow. Lysines mutated in K3E and K6E constructs are shown as sticks, and the N-terminal domain deleted in the ΔN constructs is indicated in yellow. (B) Sedimentation assay with NatA mutants. Naa15 is indicated. Note for the ΔN mutants, Naa15 runs lower than WT Naa15. The faint bands above 80 kD in these lanes is not Naa15, but rather impurities from the ribosome prep. These lanes are marked with asterisks. The Naa10 band is obscured by the ribosomal proteins in the gel.

The similarity of the ribosome affinities of methionine aminopeptidase, signal recognition particle, and NatA, as well as their shared ribosomal binding site and salt dependence for ribosome interaction led us to hypothesize that the NatA-ribosome interaction was similar to other protein interactions at the exit tunnel, and was largely dependent on electrostatic interactions. Therefore, we searched for conserved electropositive regions on the surface of NatA, which could account for this (Fig 3). We reasoned that electropositive regions would likely interact with the ribosome via L23 and rRNA. We generated a conservation map onto NatA [51] and compared that to the calculated vacuum electrostatics on the protein (Fig 3B and 3C). We focused on Naa15, as this subunit has previously been shown to mediate the interaction with the ribosome [32]. There were two notable regions that we found on the surface of Naa15 that were conserved and electropositive. One was within an N-terminal domain of Naa15, located in the first three tetratricopeptide repeats (TPR) of the protein (which we call electropositive region 1, EPR1), and the other was in a long, basic α-helix near the C-terminus of the protein (EPR2) (Figs 3 and 4A). Importantly, both EPR1 and EPR2 are on the same side of the enzyme, and would orient the active site of Naa10 toward an emerging N-terminus when bound to the ribosome. Moreover, both of these regions are dynamic in crystal structures of NatA, having high B-factors, and are often positioned in different orientations compared to the rest of the core of the complex in different structures of NatA [23, 47]. This may indicate a conformational flexibility required for these regions to properly orient themselves to interact with the ribosome.

In order to test the contribution of EPR1 and EPR2 on ribosome binding, we made a number of different mutations targeting these regions (Table 1 and Fig 4A). We mutated three lysines to glutamates in EPR1 (K3E), six lysines to glutamates in EPR2 (K6E), and combined all nine of these mutations into one construct (K9E). We also deleted the first three N-terminal TPR repeats (ΔN in Fig 4A), and also made a mutant combining this with the mutations in EPR2 (ΔN-K6E). We decided to delete the entire N-terminal domain since this entire region displayed higher conservation than most of the Naa15 subunit (Fig 3B).

**Table 1:** NatA mutants used in this study.

NatA variant	Mutations in variant
WT	N/A
K3E	K27E, K28E, K31E
K6E	K605E, K606E, K609E, K610E, K612E, K613E
K9E	K27E, K28E, K31E, K605E, K606E, K609E, K610E, K612E, K613E
ΔN	Δ1–109
ΔN-K6E	Δ1–109, K605E, K606E, K609E, K610E, K612E, K613E
K2E_A_	K216E, K217E
K2E_B_	K342E, K345E
K2E_C_	K398E, K401E

The mutations in these regions had negative effects on binding to the ribosome. Both the mutations targeting lysines in EPR1 (K3E) and EPR2 (K6E) displayed weaker binding to the ribosome than WT (Fig 4B), and combining the two regions together (K9E) displayed an even greater effect. The ΔN mutations displayed a more severe effect than K9E (Fig 4B), and thus the N-terminal region seems to be a major contributor to the interaction. This suggests that regions in the N-terminus outside of EPR1 also strongly contribute to ribosome binding, and that the electrostatic interactions predicted are not a complete description of the interaction.

**Fig 5:**
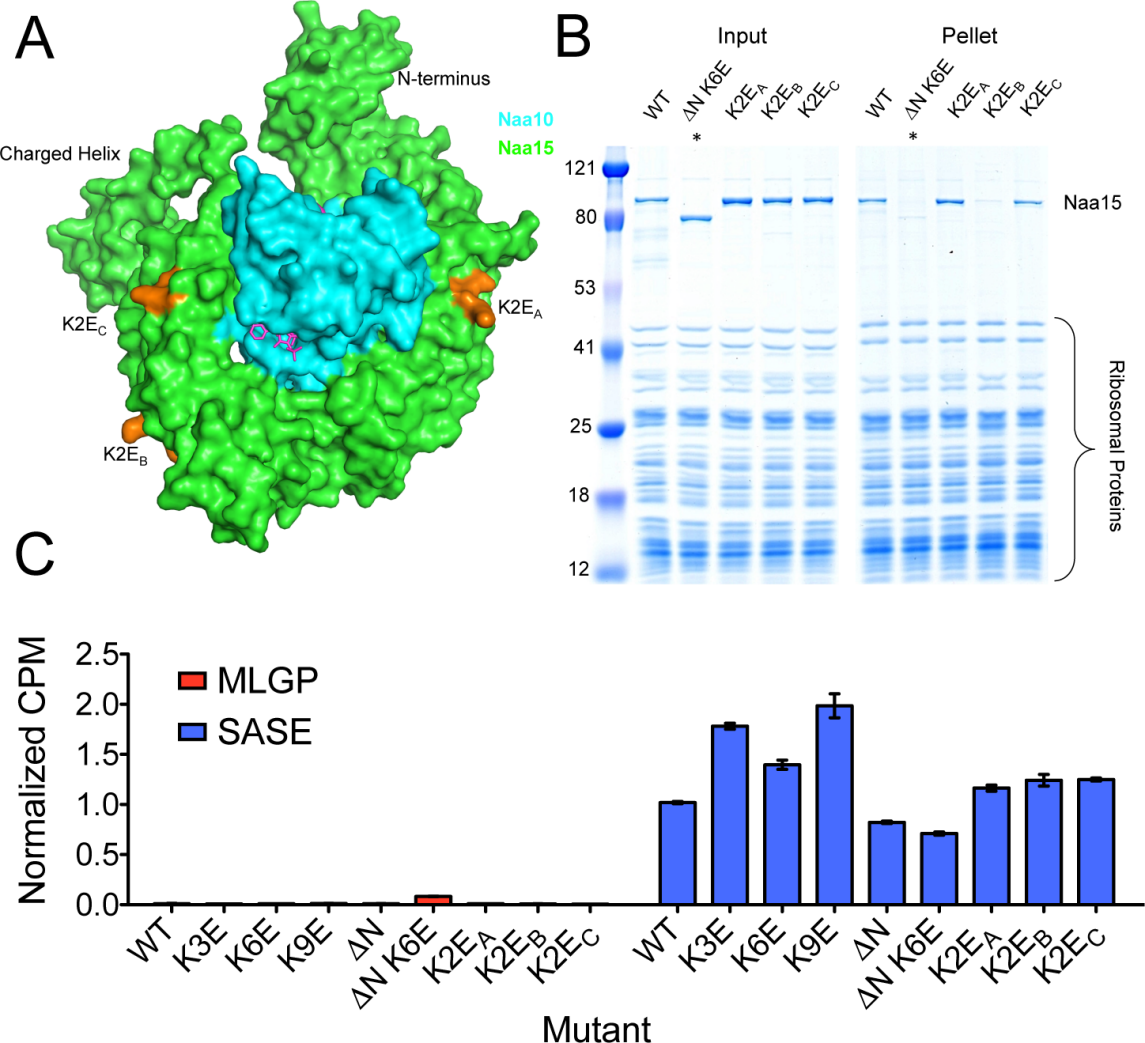
Pull down analysis of controls. (A) A surface view of the NatA complex. Mutated lysine residues are indicated in orange and labeled with their mutant name. B) Pull down analysis of the NatA mutants. Asterisks are used as in Fig 4. C) Activity analysis of mutants. SASE is a peptide corresponding to a known NatA substrate, and MLGP is a peptide corresponding to a NatE substrate (See materials and methods for full length peptide sequences). Assays were done in triplicate.

We also constructed a number of lysine to glutamate mutations in other regions of Naa15 as controls to test if disrupting other regions of positive charge on the protein would have the same effect (Fig 5A). Two of these mutants (K2E_A_ and K2E_C_, Table 1) behaved as the WT enzyme (Fig 5B), but interestingly, one did not (K2E_B_, Table 1). We do not know why the mutations of K342E, K345E in NatA (K2E_B_) are sensitive for ribosome binding, since they are poorly conserved among NatA orthologs and are not oriented in the same interface as the EPR1 and EPR2 regions we identified. Nonetheless, this data indicates that other surfaces of NatA outside of EPR1 and EPR2 also contribute to ribosome binding.

We tested all of the mutants with an activity assay to ensure that they were properly folded and found that each of the mutants retained catalytic activity within ~2-fold of the wild-type enzyme (Fig 5C). We tested the activity against two different peptides. The first, which we refer to as SASE, after the four first N-terminal residues is a strong NatA substrate [47]. The second, MLGP, is a NatE substrate, which NatA cannot acetylate [47, 52]. We used SASE as a positive control, and MLGP as a negative control for the folding of the NatA complex. Note that Naa10 is not active in the absence of Naa15, which wraps around Naa10 and repositions one of its substrate binding loops [47]. These assays indicate that all of the mutants contained a properly folded Naa15-Naa10 interaction, and that the ΔN mutant retained activity suggests that this region is not required for the Naa15-Naa10 interaction.

**Fig 6:**
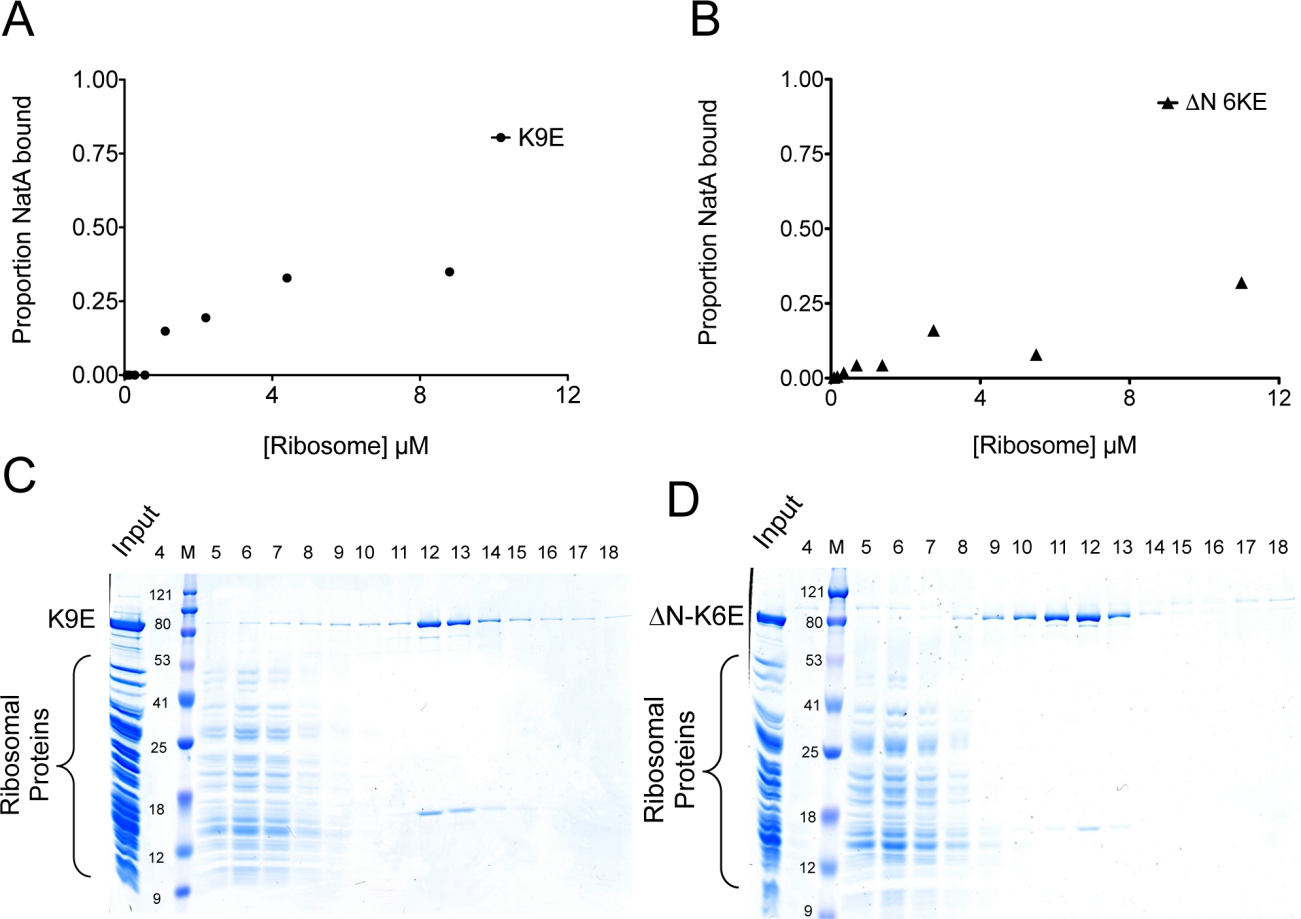
ΔN-K6E does not bind to ribosomes. (A) Binding profile of K9E and (B) ΔN-K6E. These data could not be fit to a binding curve. Compare to Fig 2B (C) Fractions of the ribosome and an excess of K9E and (D) ΔN-K6E eluting off of a Superose 6 column. Compare to Fig 1B.

After the co-sedimentation assays, we focused on K9E and ΔN-K6E (Table 1). We tested K9E to further test the hypothesis that EPR1 and EPR2 are critical for interaction with the ribosome, and tested ΔN-K6E since it displayed the most severe pulldown defect. We decided to pursue ΔN-K6E even though the ΔN mutation alone was almost as severe in ablating the interaction (Fig 4B). This was based on the weaker binding of K9E than either K3E or K6E alone, which indicates that EPR2 is important, despite the N-terminus appearing to be a more crucial region in the interaction. These mutations are predicted to highly perturb the electropositive regions on the surface of NatA (S2 Fig). We performed co-sedimentation over a range of ribosome concentrations to determine the affinity of these mutant to the ribosome. The resulting data could not be fit to a curve, yet the ribosome only began significantly pulling down ΔN-K6E at 10 μM, and did not reach above about 30% pulldown for K9E at the concentrations used. This indicates a significant decrease in affinity between the ribosome and the mutant complexes tested (Fig 6A and 6B). We also performed gel filtration with the mutants and observed weakened to no binding between the ribosome and the mutants (Fig 6C and 6D). These data indicate that EPR1 and EPR2 are important contributors to ribosome binding, and that mutations of these regions highly disrupt the interface between the ribosome and NatA. The data also suggest that the N-terminal region has other important interaction mediating residues outside of EPR1, as the ΔN-K6E mutant ablated the interaction more than the K9E mutation.

## Discussion

Here we have presented data on the direct interaction between the ribosome and NatA. Studies with purified ribosomes and NatA reveal a salt dependent interaction with a dissociation constant of about 1 μM similar to other enzymes, which bind near the exit tunnel of the ribosome [37, 38, 53]. Conservation analysis led us to identify two regions of the protein important for ribosome binding: An N-terminal region that contains a positively charged surface, and a positively charged helix near the C-terminus. Importantly, these regions are on the same side of NatA, and would orient the active site of Naa10 to face the peptide coming out of the exit tunnel. The deletion of the entire N-terminus in this work precludes us from specifically identifying the residues in this region that mediate the interaction. In addition, the finding that the K2E_B_ mutant is also defective in ribosome binding suggests that this model is still incomplete, and more work will be necessary to dissect other contributions to the NatA/ribosome interaction.

**Fig 7:**
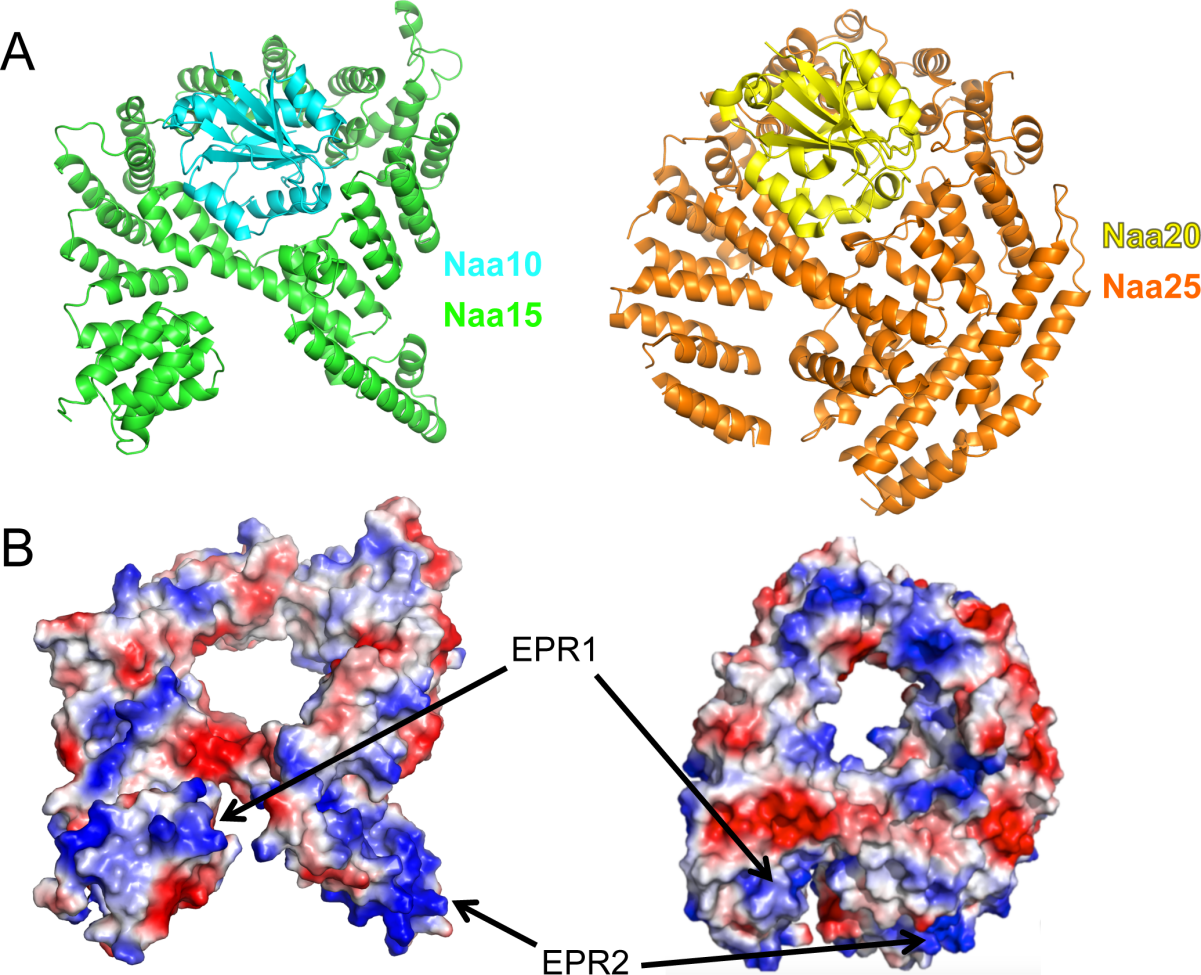
NatB has positively charged regions in the same configuration as NatA. (A) Cartoon representation of the NatA and NatB complex. Naa10 is shown in cyan and Naa15 in green. Naa20 is shown in yellow and Naa25 in orange. (B) Electrostatic surface representation of Naa15 and Naa25 with EPR1 and EPR2 on the Naa15 structure, and putative areas on Naa25 corresponding to these regions indicated with arrows.

Despite the of preliminary nature of the model, other lines of evidence from recent studies also support the notion that NatA regions EPR1 and EPR2 are important for ribosome binding. A number of structures of Naa50 bound to the NatA complex have been determined (PDB IDs: 4XPD, 4Y49, 4XNH) [54], and Naa50 is positioned in such a way that its active site would similarly face the peptide emerging from the exit tunnel, which is consistent with the model proposed here. NatA was also recently crystallized with its binding partner HYPK, and that complex would still allow for ribosome binding via EPR1 and EPR2, as they are not part of the interface between the two proteins [23]. In addition, the NatB structure, which was also recently reported, shows a similar molecular “nest” of Naa25 around Naa20 [55]. The TPR repeats of Naa25 are arranged differently than Naa15, particularly in the region where the ribosomal binding determinants are found in NatA. Notably, however, there are two regions on Naa25, which contain positively charged regions in the same positions as Naa15 (Fig 7). This suggests that the mode of ribosome binding could be similar between the different NATs. An open question in the field is whether the NATs compete for the same binding site on the ribosome, or if they can bind simultaneously. These results, along with the fact that all of the NATs are substoichiometric with the ribosome in the cell [56], point to NATs competing for the same binding sites on the ribosome and quickly sampling the peptides being translated.

These studies raise a number of questions about the interaction of NatA and the ribosome. Does NatA compete with the other NATs, and the other N-terminal processing enzymes, which bind to the exit tunnel? For NatA in particular, methionine aminopeptidase needs to act on iMet before NatA can acetylate the N-terminus [20]. Whether NatA binds simultaneously with MAP and if the presence of one increases the affinity of the other is an open question. The stoichiometry of the complex is also unknown. We believe the stoichiometry of the NatA:ribosome complex to be 1:1, based on a number of factors. First, in pull down assays, the band intensity indicated a 1:1 stoichiometry of the Naa15 band with the rest of the ribosome bands (Fig 4B, WT pellet sample). When we compared the band intensity of the Naa15 and the first two ribosomal proteins around 41 kD, we obtained a ratio of 1.13, and 1.28, which agree best with a 1:1 stoichiometry. Second, the curve fit very well to a 1:1 model, with an R^2^ value of 0.94. Despite this reasoning, we cannot definitively conclude that the binding is 1:1, and more studies will be necessary to conclusively determine the stoichiometry.

A related question is the importance of NatA dynamics for ribosome binding. The N-terminal processing enzymes are highly dynamic, and, as mentioned, substoichiometric to the ribosome in the cell. Although NatA can bind to the non-translating ribosome, there may be an ordering only upon binding to ribosomes that are translating. It may be that NatA binding to the ribosome is modulated and/or increased by cognate peptide emerging from the exit tunnel. Indeed, SRP affinity for the ribosome increases significantly when there is a peptide emerging from the tunnel [35]. Trigger factor also displays increased affinity and slower on/off kinetics for the ribosome in the presence of a nascent peptide [53, 57]. These findings may also contribute to the therapeutic targeting of NatA by disrupting its interaction with the ribosome. To date, it has been a challenge to find specific and potent inhibitors for acetyltransferases, even though they are attractive drug targets [58]. These studies point to the targeting of Nat interaction with the ribosome as an alternative to targeting the catalytic pocket of Nat enzymes.

Together, these studies provide the first molecular framework for understanding how NatA interacts with the ribosome for co-translational protein N-terminal acetylation. These findings have implications for how other NATs may interact with the ribosome and will facilitate further research into the significance of co-translational N-terminal acetylation for a plethora of biological processes.

## Supporting information

S1 Fig Uncropped gels from Fig 2. (A) Uncropped gel from Fig 2A. Note that the gel is flipped horizontally in Fig 2A (B) Uncropped gel from Fig 2C.
(TIF)

S2 Fig Electrostatic potential maps of NatA mutants. Electrostatic potential map of NatA mutations. Blue areas represented regions which are electropositive, and red areas represent regions which are electronegative. Electropositive region 1 (EPR1), and electropositive region 2 (EPR2) are indicated for wild-type NatA (A). Electrostatic potential map of K9E (B). Electrostatic potential map of ΔN-K6E (C).
(TIF)
