# Supplementary figures and images for: Probing the interaction between NatA and the ribosome for co-translational protein acetylation
Source: PLoS One. 2017 Oct 10;12(10):e0186278. doi: 10.1371/journal.pone.0186278 (PMC5634638; doi:10.1371/journal.pone.0186278)

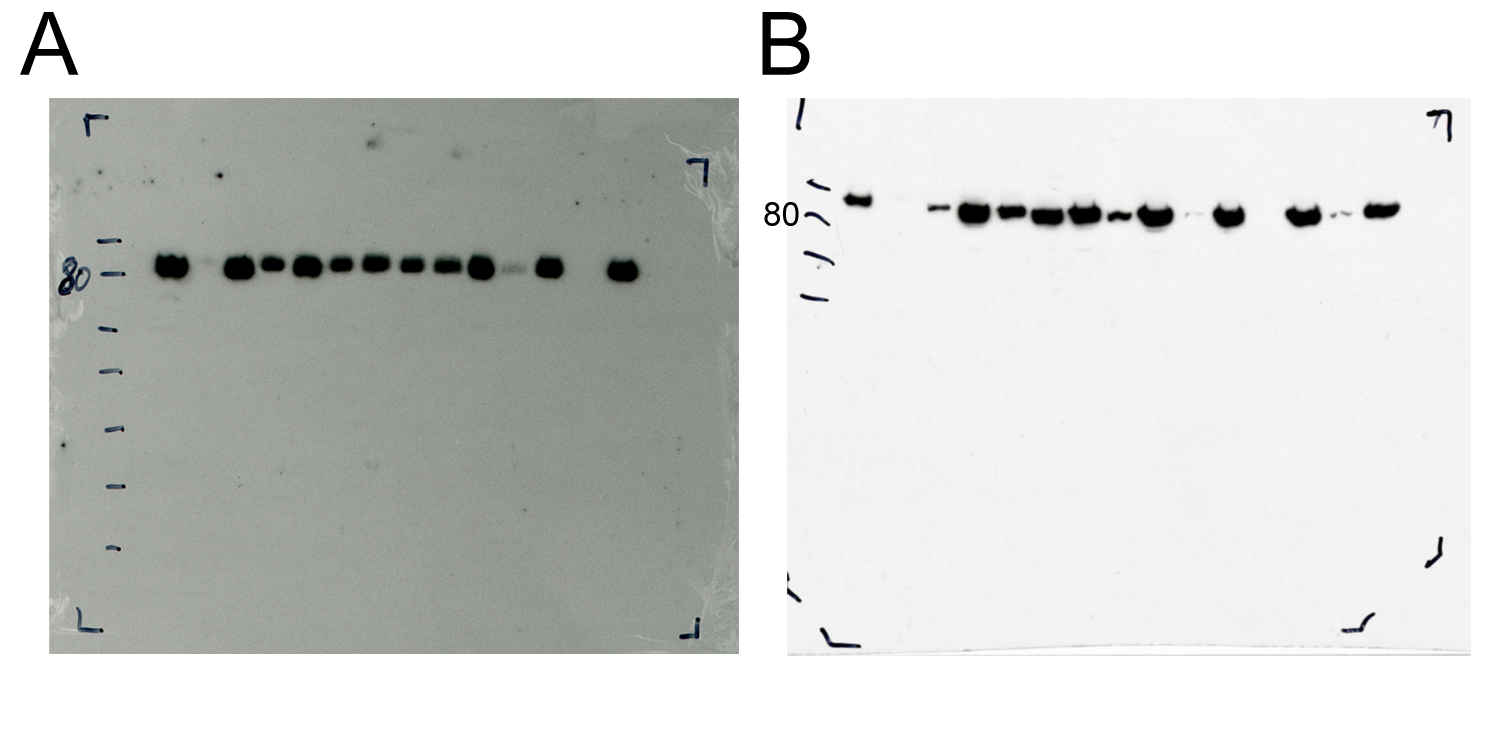

Supplement: S1 Fig — (A) Uncropped gel from Fig 2A. Note that the gel is flipped horizontally in Fig 2A (B) Uncropped gel from Fig 2C. (TIF) [file pone.0186278.s001.tif]

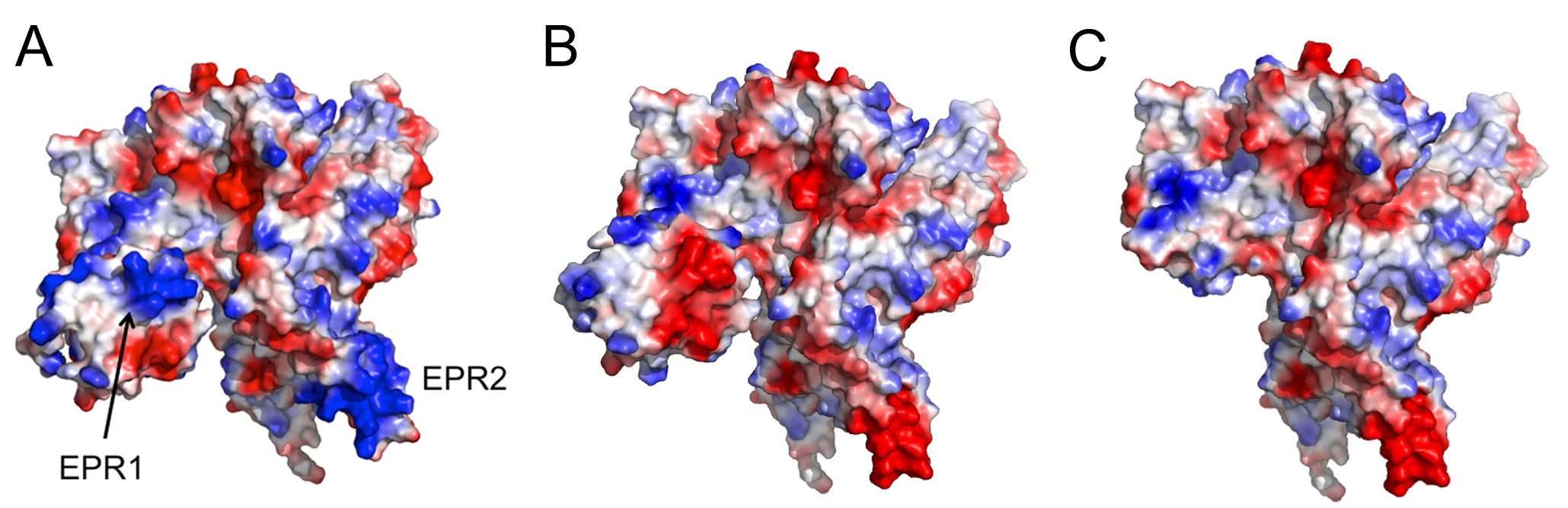

Supplement: S2 Fig — Electrostatic potential map of NatA mutations. Blue areas represented regions which are electropositive, and red areas represent regions which are electronegative. Electropositive region 1 (EPR1), and electropositive region 2 (EPR2) are indicated for wild-type NatA (A). Electrostatic potential map of K9E (B). Electrostatic potential map of ΔN-K6E (C). (TIF) [file pone.0186278.s002.tif]
